# Investigating the spatial effects of zonal factors on road traffic speed variability during peak hour

**DOI:** 10.1371/journal.pone.0340583

**Published:** 2026-01-08

**Authors:** V. A. Bharat Kumar Anna, Sai Chand, Abdulmajeed Alsultan, Vinayak Dixit

**Affiliations:** 1 Vignan’s Foundation for Science, Technology and Research, Guntur, Andhra Pradesh, India; 2 Transportation Research and Injury Prevention (TRIP) Centre, Indian Institute of Technology Delhi, New Delhi, India; 3 Department of Civil Engineering, College of Engineering, Prince Sattam Bin Abdulaziz University, AlKharj, Saudi Arabia; 4 Research Centre for Integrated Transport Innovation (rCITI), School of Civil and Environmental Engineering, University of New South Wales, Sydney, New South Wales, Australia; Chang'an University, CHINA

## Abstract

While traffic speed is a popular metric for evaluating road network efficiency and safety, its application is often limited. Studies typically focus on short stretches of road, specific design features, or particular highways, and even then, only during certain timeframes. This might be due to a lack of comprehensive traffic data. Further, analysing entire road networks and landuse patterns at a macro-level has been less common. Therefore, the study’s objective is to understand the factors influencing traffic speed variations at a zonal level using crowdsourced pervasive traffic data. Crowdsourced Speed (CS) data was collected every 10 minutes for thirty consecutive days in the Central Business District (CBD) area in Sydney, Australia. Data on road network characteristics, land use, public transportation, socioeconomic variables, and travel behaviour at the zonal level were gathered from various sources for analysis. Principal Component Analysis (PCA) based local regression models were developed to understand the variables influencing speed variation at the zonal level for both morning and evening peak hours. Two Geographically Weighted Regression (GWR) models were developed to examine spatial variability in the coefficient of variation (CoV) of speed. The spatial distribution of local *R²* indicates that it ranges from 0.102 to 0.57 for the morning peak hour and from 0.37 to 0.54 for the evening peak hour. The variable coefficients indicate that compared to the morning peak-hour model, the evening peak-hour model exhibits greater consistency and statistical significance (at the 0.10 level) across most zones in explaining the coefficient of variation (CoV) of speed. For the evening peak hour, density, private vehicle travel, commercial activity, land use diversity and income, and road network connectivity factors are significant in explaining the variability. Analysing speed variability at the zonal level across a large network will enable planners and engineers to prioritise zones for traffic improvements.

## Introduction

Road network performance is commonly assessed through travel time and/or speed at various levels, including individual links [1,2], zones [3], or the entire network [4–6]. The two measures, travel time and speed, are interrelated, i.e., as speed increases, the travel time decreases and vice versa. However, the significance of the measures varies depending on the type of application. Travel time analysis is commonly employed in transportation planning, operations, and traffic monitoring [6–9]. Conversely, speed analysis is prevalent in safety assessments and congestion indicators [1,2,4,5,10]. Here, understanding traffic speed is two-fold, i.e., on the one hand, slow-moving traffic (congestion) diminishes road network efficiency by prolonging travel time and increasing fuel consumption and emissions [11]. On the other hand, high speeds and speed fluctuations significantly contribute to road crash fatalities [11–13]. Statistics from India, Australia, and Europe highlight speeding as the leading cause of road fatalities, accounting for 75.2%, 41%, and 30% of fatalities, respectively [14–16]. However, some studies suggest that crashes are more likely when network speeds are lower due to congestion [17]. Thus, analysing and modelling speed and its variability is crucial from both traffic congestion and safety perspectives.

Speed variables in different forms were widely used as predictor variables to quantify their effect and impact on crashes [18,19]. Traffic speed exhibits variability across a road network due to several factors, including road type, traffic conditions, environmental elements, land use, driver attributes, geometric design, terrain, etc. [13]. However, comprehensive research into the factors affecting speed variations across all road segments within specific categories at the zonal level remains lacking. Examining speed variations at the zonal level within a city aids transportation planners in identifying zones with pronounced speed fluctuations and understanding the underlying causes to implement necessary mitigation strategies. Moreover, zonal-level analysis facilitates the identification of high-risk zones concerning crashes attributed to speed and other zonal attributes. Here, the primary challenge lies in accessing quality and timely data at a finer resolution.

In transportation research, the duration of data collection stands out as a crucial consideration when analyzing traffic data. This is because traffic data, particularly speed data, significantly varies depending on the time of day, day of the week, between weekdays and weekends, and month of the year [11,20]. However, the choice of data collection period largely hinges on the researcher’s interests. Safety studies primarily aim to monitor speeds under daylight and dry weather conditions [21,22], over an extended period, typically exceeding a month [23], to analyze or predict crashes and crash rates. While some explored speed differentials between peak and off-peak hour of a week to investigate the underlying factors [3,24]. Yet, when dealing with large road networks, existing equipment like fixed sensors and GPS-equipped vehicles may not be suitable for capturing traffic data over extended periods and diverse times of the day. Fixed sensor installations are constrained to specific locations, incurring high maintenance and data extraction expenses [24]. GPS-equipped vehicles, typically used as probe vehicles, are more suited for smaller-scale studies such as specific highways, road sections, or smaller networks [21,25]. However, their feasibility for larger study areas depends on factors like sampling rate, time, and cost. In this context, Crowdsourced Speed (CS) data derived from probe vehicles and mobile phones has gained popularity for its ability to continuously gather location-based travel information, including travel times, speeds, incidents, and more, across larger networks without the need for on-site equipment. Consequently, Crowdsourced pervasive traffic data has emerged as a viable alternative to traditional field data collection methods [4,26]. Moreover, studies have assessed the effectiveness and reliability of SC data for scientific research purposes [26,27].

Thus, this study aims to explore the spatial effects of zonal factors on road traffic speed variability during peak hour using Crowdsourced speed (CS) data for a part of the Sydney Greater Metropolitan Area, Australia. The remainder of the paper is structured into six sections. Section 2 provides a brief overview of previous studies concerning the determinants of speed variations. Section 3 presents the study area and data collection. Section 4 delineates the methodology. Section 5 offers the preliminary analysis, while Section 6 presents the model development and results. Lastly, Section 7 delves into a discussion of the results, and Section 8 concludes with implications and avenues for future research.

## Background

Traffic speed has been extensively utilised as a dependent variable to explore the underlying factors affecting congestion and speed fluctuations. Garber and Gadiraju, (1989) investigated the influence of traffic and road geometry factors on speed variation [28]. They found that design speed and highway type were significant factors, whereas traffic volume did not significantly contribute to explaining speed variance. Ericsson, (2000) examined the influence of different street types, drivers, and traffic conditions on speed variability in urban roads [29]. The study found that street type has a greater influence on the driving pattern. Compared to off-peak hours, peak-hour conditions are likelier to have lower mean speeds and average deceleration levels. In the comparison of gender, men had higher acceleration rates and always tended to travel fast. Fitzpatrick et al., (2005) developed road category-specific models to understand various factors influencing the speed on different road functional hierarchies [30]. The study found that only posted speed limit and access density as significant variables that influence roadway speeds. Quddus, (2013) investigated the relationship between speed variables, i.e., mean speed and speed variation (standard deviation), and crash rates across several road segments [18]. The study found that, compared to mean speed, speed variation is statistically significant and positively associated with crash rates.

Li et al., (2016) investigated the effect of rainfall intensity and traffic characteristics on the traffic speed variability on urban roads [31]. The study found that the speed variability, as measured with the coefficient of variance, increased in rainy conditions compared to dry weather conditions. Silvano and Bang, (2016) analysed the impact of changed posted speed limits along with the road characteristics on free-flow speed in urban areas [32]. Their investigation revealed that decreased posted speed limits led to notable reductions in mean speed and speed variance. Further, road characteristics such as carriage width, road environments, and the presence of on-street parking and sidewalks were found to influence the free-flow speed. Thiessen, (2016) developed road category-specific models to explore the factors affecting operating speed on urban roads [33]. The study revealed that roads characterized by longer segments, wider medians, and bicycle facilities exhibited increased operating speeds, while factors like bus stops, object/tree density, and access points were associated with decreased operating speeds.

Nair et al., (2019a) used a traffic speed-based Congestion Index (CI) to analyse the spatial dispersion of traffic congestion of multiple cities across the world [4]. The study revealed that the variations in congestion patterns have strongly linked to macroeconomic factors such as population density, GDP per capita, pollution emissions, and road network structure. Zhong et al., (2021) explored the spatial heterogeneity of urban built environments on the mean speed of the road segments through geographically weighted regression (GWR) [34]. They identified several factors, including the number of bus stops, distance to the nearest school or intersection, occupancy rates of taxis, and speed limits, as significant contributors to mean speed variations. Similarly, Nian et al., (2021) investigated the effect of segment-based urban built environments through points of interest (POIs) on road travel speed [35]. Employing a spatial heterogeneity analysis with the GWR model, they found that factors such as bus stops, healthcare services, recreational facilities, parking entrances and exits, residential and commercial areas significantly affected travel speeds. Martinelli et al., (2022) developed two distinct models, one for the mean speed and another for the standard deviation of speed, and the study revealed that segment length, lane width, median presence, density of bus stops and pedestrian crossings, presence of curbs and sidewalks, and land use types adjacent to roads significantly contributed to speed Variability [36]. Rahman et al., (2022) investigated macroscopic factors of urban areas that contribute to traffic congestion [37]. Analysing data from multiple metropolitan areas/cities in the United States, found that population size, per capita income, and employment concentration as key factors contributing to traffic congestion. Additionally, the study highlighted that non-car-based mode share, highway infrastructure, community structure, urban density, and socioeconomic factors play roles in alleviating congestion. Further details of the above studies are presented in Table 1.

**Table 1:** Summary of literature on analysing factors influencing speed or speed variability on urban roads.

Author	Data collection	Duration of data collection	Study stretch/network	Speed variables	Independent variables	Location/Country
Garber and Gadiraju, (1989) [28]	Leupold & Stevens traffic data recorder	24 hours on weekdays	Interstates, arterials, and rural major collectors	Speed variance	Road geometry-related	Virginia
Ericsson, (2000) [29]	GPS instrumented vehicle	Peak and off-peak hours	A route with multiple road segments	µ & σ of speed	Street types, traffic conditions, and drivers	Sweden
Fitzpatrick et al., (2005) [30]	Spot speed	Weekday under free-flowing conditions	79 tangent sections, includes local, collector, & arterial roads	85^th^ percentile speed	Roadside, road geometry, access points, & traffic control related	United States
Quddus, (2013) [18]	Highways Agency	Average hourly speed data for one year	13 motorways & 17 trunk roads	µ & σ of speed	Traffic & road geometry-related	London, United Kingdom
Wang et al., (2014) [38]	GPS instrumented taxis	Five weekdays in peak & off-peak hours	Arterial road network	Mean speed (µ)	Geometric, traffic control & traffic volumes related	Shanghai, China
Li et al., (2016) [31]	Video traffic detector	Six months	2^nd^ and 3^rd^ lane of Gloucester road	CoV of speed	Traffic and weather-related	Hong Kong
Silvano and Bang, (2016) [32]	Spot speed	100 observations per site on weekdays under free-flow conditions	118 sites that include arterial, main, & local roads from 11 cities	Mean speed (µ)	Posted speed limit, road characteristics	Sweden
Thiessen, (2016) [33]	Fixed sensors	Four years	Arterials, collectors, & other roads	85^th^ percentile speed	Onroad, roadside & traffic related	Edmonton, Canada
Nair et al., (2019a) [4]	Google (SC)	40 days	29 cities around the world	Congestion Index (CI)	Macro economic characteristics related	NA
Pan et al., (2020) [3]	NA	2- weeks for evening peak hours on weekdays	All road types in central urban area	Traffic state index	Land use, road & public transport related	Shanghai, China
Zhong et al., (2021) [34]	GPS instrumented taxis	Two-hour period for three days	10 km road stretch	Mean speed (µ)	Onroad & roadside-related	Shenzhen, China
Nian et al., (2021) [35]	GPS instrumented taxis	3 days	Central City road network	Mean speed (µ)	Urban built environment related	Chongqing, China
Martinelli et al., (2022) [36]	Spot speed	Weekday under free flow conditions	37 locations, 24.7 km length of urban road network	µ & σ of speed	Onroad, roadside land use, & traffic related	Brescia, Italy
Rahman et al., (2022) [37]	Secondary data source	NA	100 metropolitan areas	Traffic congestion indices	Structural, socio-economic & behavioral related	USA
Phan and Truong (2024), [39]	NA	Peak hours	Greater Melbourne	Mean speed (µ)	Demographics, land use, and journey-to-work	Australia
Xiao et al., (2024) [40]	Bluetooth and loop detectors	1 year	Greater Melbourne metropolitan region	Mean speed (µ)	Road network, socio-economic, travel, and built-environment	Australia

In summary, a substantial body of research has been dedicated to understanding the factors influencing speed and its variability. As outlined in Table 1, most of these studies focused on specific traffic conditions, such as peak hours, free-flow scenarios, weekdays, or weekends, reflecting each study’s unique research objectives. A predominant emphasis has been placed on link-level characteristics, focusing on on-road or roadside features. However, recent literature has highlighted the growing relevance of zonal-level influences in understanding broader travel behavior and safety outcomes [41]. While limited in the context of speed variability studies, several investigations in the crash modeling domain have employed zonal-level explanatory variables, including land use mix (entropy), road network structure (e.g., meshedness coefficient, completeness index), and socioeconomic indicators [40–46]. These factors are particularly important for understanding spatial variation in road user behavior and traffic safety outcomes at mesoscopic or macroscopic levels. Further, few explored the association between the traffic congestion index and the likelihood of having fatal crashes at the zonal level [39]. Thus, zonal level factors are particularly important for understanding spatial variation in road user behavior and traffic safety outcomes at mesoscopic or macroscopic levels. Nevertheless, only a few studies have analysed speed and its variability at the zonal-level [3]. In this context, this study aims to explore the influence of various zonal-level factors, including the road network on speed variability during peak hours throughout the weekdays in the vicinity of the Central Business District (CBD) of Sydney, Australia.

## Study area and data collection

### Study area

**Fig 1:**
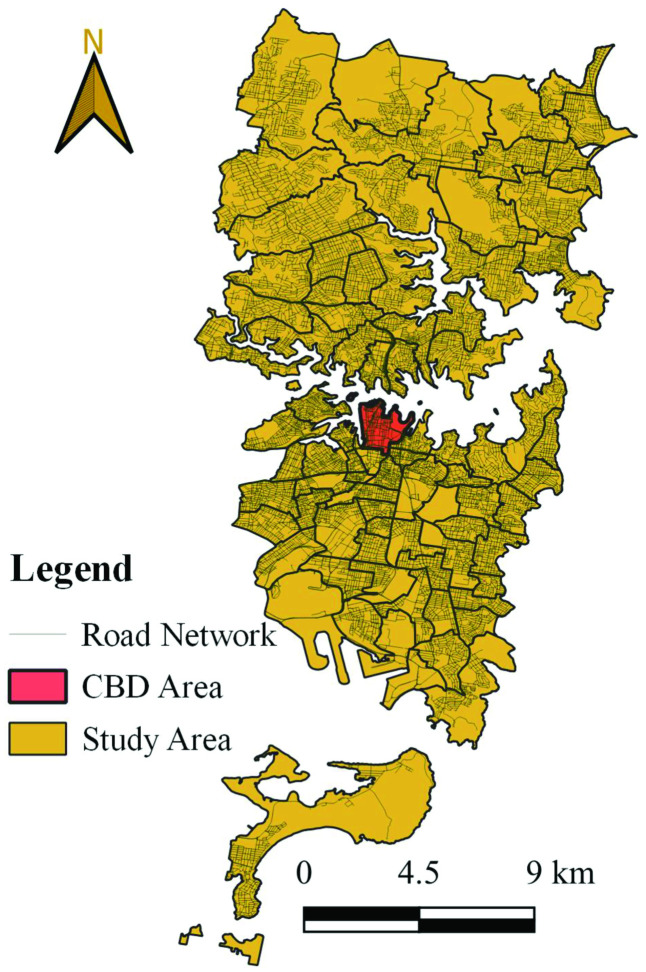
Geographical representation of the study region in Sydney, Australia. The figure presents a schematic of the road network, and highlighting the Central Business District (CBD) in the study region.

The study area in this research encompasses the vicinity of the CBD of Sydney, Australia, stretching from Narrabeen in the north to Maianbar in the south. This area attracts a significant portion of Sydney’s population daily, drawn by the presence of office buildings, historical landmarks, iconic structures, and picturesque landscapes. Additionally, populated districts such as Bondi Junction, Mascot, and Chatswood are included within the study region. The entire study region is subdivided into 82 zones according to the Statistical Area Level-2 (SA2) [47]. Fig 1 illustrates the selected study area located in Sydney, Australia. According to the latest TomTom traffic index, commuters in Sydney experience the worst congestion during weekdays between 8 a.m. to 9 a.m. and 5 p.m. to 6 p.m. [48]. During morning peak hours on a typical workday, the average number of trips towards the CBD area reaches approximately 100,000 trips per hour, accounting for 47% of the total daily trips to the CBD [49,50].

### Data collection

CS data were collected at a frequency of 10 minutes over a span of thirty consecutive days, i.e., from 1^st^ June 2018–30^th^ June 2018, through the Google Speed Application Programming Interface (API). Google utilizes anonymized location data collected from GPS-enabled devices, including smartphones and in-vehicle navigation systems, to estimate real-time traffic parameters such as speed and travel time. This data is primarily sourced from users who have enabled the “Google Maps” service or the “My Location” functionality on their devices [51]. The collected information is processed through various Application Programming Interfaces (APIs) developed by Google, which support advanced functionalities including spatial data analysis, predictive modeling, and data integration, subject to user consent for data sharing [27]. A key output of these APIs is the estimation of traffic speed, wherein the system computes representative speed values for individual road segments based on aggregated and temporally synchronized crowdsourced data. This enables dynamic, high-resolution insights into traffic conditions throughout the day. In this study, the CS data corresponds to approximately 32,000 locations covering various road categories passing through Sydney’s CBD. Here, each location represents a georeferenced point along a link (road segment) where data such as speed, position, and timestamp were recorded during a vehicle’s trajectory. A single road segment can have multiple such points, depending on the sampling frequency of the GPS device and the speed of the vehicle traversing that segment. The dataset used in this study reflects actual vehicle movements, and the spatial coverage is determined by the specific routes taken by vehicles during the data collection period. For more details on the data collection procedure, readers may refer to Nair et al., (2019a) [4]. Prior to analysis, the CS data is processed to clean the data for missing entries, duplicates, and outliers. First, records with missing values were systematically identified and removed to ensure the completeness of essential variables. Next, duplicate entries were detected via unique ID matching and removed to prevent data redundancy. Finally, outliers were removed from the data through quartile analysis. Additional data, including road network attributes, public transit information, socioeconomic indicators, land use characteristics, and zonal details, were also collected from diverse sources.

#### Road network data and metrics.

The road network within the study area was retrieved from OpenStreetMap (OSM) using OSMnx and Rapidex [52,53]. In the road network, OSM defines the road type into seven categories based on the functional road hierarchy. The road categories are motorways, trunk roads, primary roads, secondary roads, tertiary roads, residential roads, and unclassified roads. Various characteristics of the road network, such as road length, edge count, node count, and lane count for each road category within each zone, were determined using GIS tools. The road network is characterized by various metrics, including edge and node density, meshedness coefficient, completeness, and entropy. The meshedness coefficient (α) is the ratio of the actual number of circuits in a network/zone to the maximum possible number of circuits. The completeness (ρ) index indicates how well a road network captures all the roads present in the given zonal area, whereas entropy (*H*) defines the heterogeneity [54]. These metrics were computed using Equations 1–3 [54].


$$\alpha_{j}=\frac{m_{j}-n_{j}+\text{1}}{\text{2}n_{j}-\text{5}}$$ (1)

$$\rho_{j}=\frac{n_{j}}{\left({m^{\text{2}}}_{j}-m_{j}\right)}$$ (2)

$$H(X_{j})=\;-\sum_{i=\text{1}}^{I}p_{i}\;{log}_{\text{2}}(p_{i})$$ (3)

Here, m and n are the number of edges and nodes in *j*^*th*^ zone, respectively. In equation 3, $H(X_{j})$ is the entropy of road in *j*^*th*^ zone; *i* is the number of edge/road categories within a zone, and $p_{i}$ is the proportion of edges that fall in *i*^*th*^ road type.

#### Public transit data.

The static General Transit Feed Specification (GTFS) data corresponding to bus transit was obtained from the [55]. This data includes bus schedules along with their routes, bus stop locations, and operating frequency.

#### Land use, socioeconomic, and travel characteristics.

The zone information, land use attributes, and socioeconomic and travel characteristics were sourced from the Australian Bureau of Statistics [47]. Land use was classified into eight attributes based on activity type using mesh blocks for each zone. By analysing these mesh blocks, the percentage of land covered by each attribute was determined for each zone. Additionally, similar to the entropy of roads, the entropy of land use for each zone was calculated using Equation 3. The socioeconomic characteristics include income, age, employment, mode of transport, vehicle ownership per dwelling, etc. The travel characteristics include the percentage of people travelling by bike, car, bus, train, tram, taxi, truck/commercial vehicles, etc. For the analysis, these modes were grouped into public transport, private transport, and other modes of transport. Socio-demographic and travel behavior variables primarily characterize the residential population; their inclusion in this study serves to capture latent zone-level dynamics that may influence traffic flow indirectly. In dense urban areas such as CBDs, these variables can reflect broader land use, accessibility, and travel behavior trends, even if the road users are not exclusively local residents. From the delineated zonal boundaries, it is evident that the CS data is distributed across 82 zones. The raw and processed data pertaining to land use, socioeconomic, road network, and travel characteristics have been made publicly available in an open data repository. Interested readers may refer to Anna et al., [56] for further details.

## Methodology

Global regression models like Ordinary Least Square Regression (OLS) are commonly employed to understand the factors impacting the dependent variable and/or to forecast their effect. However, due to numerous reasons, this approach may yield counterintuitive estimates. One such reason is the assumption in OLS regression models that the independent variables are homogeneous across the space, which may not hold true in all the scenarios. For instance, in the zonal, city, or country-level analysis, the variables often vary spatially, leading to spatial heterogeneity in the data [3,57]. Here, spatial heterogeneity refers to variations in the data across different spatial locations, and these variations may also be influenced by neighborhood locations, known as spatial autocorrelation [3]. In this context, researchers have explored various strategies that account for spatial heterogeneity and spatial autocorrelation by allowing some or all variables to vary. Few researchers used random parameter models [43,58,59], latent class models [43], while few others used Bayesian spatial models [60] to address the spatial heterogeneity. The GWR model is a special type of regression that accounts for spatial heterogeneity in the sampled data, and this approach has been widely used in transportation research, i.e., to understand factors influencing crash frequency [57], mode choice [42], travel speed/time [34], and travel activity/traffic states [3,61]. Therefore, this study employs the GWR model to examine the diverse factors influencing speed variability in the study region. The formulation of the GWR model is as follows:

Consider a fundamental regression equation, also known as the global regression model is expressed as


$${\hat{y}}_{i}=\;\beta_{\text{0}}+\sum_{k}\beta_{k}x_{ik}+\varepsilon_{i}$$ (4)

In equation 4, ${\hat{y}}_{i}$ represents the estimated value of the dependent variable for observation *i,* while $\beta_{\text{0}}$ stands for the intercept, $\beta_{k}$ signifies the parameter estimate for variable *k,* $x_{ik}$ represents the independent variables and denotes the value of the *k*^*th*^ variable for observation *i*, and $\varepsilon_{i}$ represents the error term. The GWR model expands upon the concept of global regression to local regression, wherein a distinct regression equation is constructed for each observation. These equations are calibrated using varying weights contingent upon the location of the observation point [62]. Thus, the expression for GWR is articulated as follows:


$${\hat{y}}_{i}=\;\beta_{\text{0}}(u_{i},v_{i})+\sum_{k}\beta_{k}(u_{i},v_{i})x_{ik}+\varepsilon_{i}$$ (5)

In equation 5, $\left(u_{i},v_{i}\right)$ denotes the location coordinates of the i^*th*^ observation point. In GWR, the key assumption is that observation points in close proximity exert a stronger influence on their respective parameter estimates compared to those further away. Each observation point in the dataset may represent either a point location or a polygon within the geographical space. Consequently, the weights assigned to the observation points are based on the distance decay function. In this context, the weight estimation process resembles that of the weighted least squares regression approach, but it is adjusted according to the location $\left(u_{i},v_{i}\right)$ of observation point *i* relative to others. The weight estimation takes the form expressed as follows


$$\hat{\beta }\left(u_{i},v_{i}\right)={\left(X^{T}W\left(u_{i},v_{i}\right)X\right)}^{-\text{1}}X^{T}W\left(u_{i},v_{i}\right)y$$ (6)

In Equation 6, the elements in bold represent a matrix, $\hat{\beta }$ denoting the estimated β, and $W\left(u_{i},v_{i}\right)$ which is also termed as W(i) is an *n *×* n* matrix. In this matrix, off-diagonal elements are zero, while diagonal elements denote the geographical weight for each of the *n* observed data for regression point *i*. This weight matrix constitutes a weighing scheme where $w_{ij}$ is determined as a continuous function of $d_{ij}$, which represents the distance between points *i* and *j*. Two commonly used weighing schemes are Gaussian and Bi-square kernels [62]. The functional forms of Gaussian and Bi-square kernels are expressed as shown in Equations 7 and 8, respectively.


$$w_{ij}=\text{exp}[-\text{1}/\text{2}{\left(d_{ij}/b\right)}^{\text{2}}]$$ (7)

$$w_{ij}={\left[\text{1}-{\left(d_{ij}/b\right)}^{\text{2}}\right]}^{\text{2}}\;if\;d_{ij}<b,\;else\;\text{0}$$ (8)

In equations 7 and 8, d represents the distance between points *i* and *j*, and b denotes the bandwidth. The bandwidth is the key controlling parameter and can be specified by either a fixed distance (fixed bandwidth) or a fixed number of nearest neighbors (adaptive bandwidth). An optimal bandwidth can be obtained by minimizing the model’s goodness-of-fit diagnostic, like cross-validation (CV) score or Akaike Information Criterion (AIC). For more detailed information, interested readers may refer to Fotheringham et al., (2002) [62].

## Preliminary analysis

The examination of CS data revealed that the dataset spanning 30 consecutive days encompasses both weekdays and weekends. This study only focuses on analysing the factors affecting travel speed during weekdays because the trip-making patterns and congestion distributions will exhibit higher and differing trends during weekdays compared to weekends [63,64]. Table 2 provides descriptive statistics for the entire month’s CS data, segregated for weekdays by each road category at a specific time, namely 8:30 a.m., which corresponds to the morning peak hour.

**Table 2:** Descriptive statistics of CS data corresponding to the functional road hierarchy.

Road categories	Statistics of speed (km/h) on Weekdays		
	Mean	Std. Dev.	Range
Motorway	42.85	18.6	103
Trunk road	34.13	15.7	89
Primary road	32.1	13.7	84
Secondary road	29.2	11.6	81
Tertiary road	27.25	9.86	82
Residential road	24.95	8.77	77
Unclassified road speed	20.3	9.54	79

**Fig 2:**
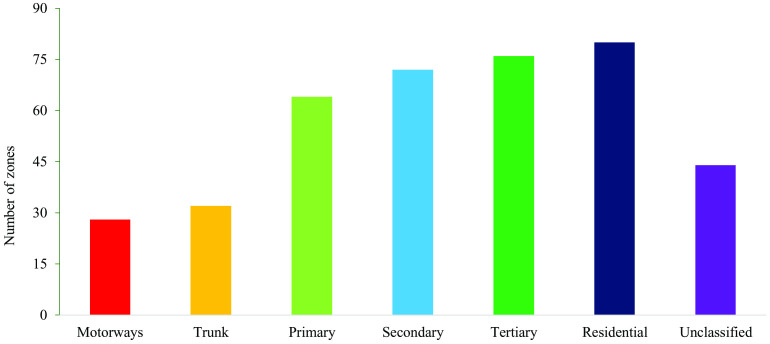
Distribution of CS data by functional road hierarchy and zones in the study region. The bar chart illustrates the distribution of data availability across different road categories and zones.

The table indicates that roads with higher hierarchies exhibit greater speeds and fluctuations. Here, the “road hierarchy” refers to the classification of roads based on their functional importance in urban mobility, where higher-order roads such as motorways and trunk roads generally support higher travel speeds and lower accessibility. This classification is relevant because it directly relates to the expected speed characteristics within each zone. Examining the distribution of CS data across road categories and zones in the study area (refer to Fig 2), it is evident that the majority of CS data corresponds to primary, secondary, tertiary, and residential roads, which span a larger number of zones (with a minimum of 64 zones covered) compared to other road categories.

This study focuses on analyzing CS data pertaining to 64 zones on primary roads during weekdays. The rationale for selecting primary roads in our analysis is based on the road hierarchy and its functional role within the transport network. Lower-hierarchy roads, such as residential and local streets, typically serve high-access functions but carry relatively minimal traffic volumes. Their primary role is to channel vehicles from neighborhoods and local areas toward higher-hierarchy roads. In contrast, higher-hierarchy roads including motorways, trunk roads, and primary roads accommodate significantly higher traffic volumes and serve as the main conduits for inter- and intra-urban mobility. From a research perspective, the focus is therefore more meaningful on higher-hierarchy roads, since they not only carry the majority of traffic generated from lower-hierarchy roads but also present greater implications for safety, capacity, and policy. Thus, among higher-hierarchy roads, primary roads were chosen in particular because they provided adequate data representation and coverage across the study zones, as illustrated in Fig 2. Additionally, the mean speed and Coefficient of Variation (CoV) of speed data for primary roads on weekdays were examined for the entire month. The CoV was calculated using Equation 9, where σ represents the standard deviation of speed and µ denotes the mean speed.


CoV= σ/μ (9)

**Fig 3:**
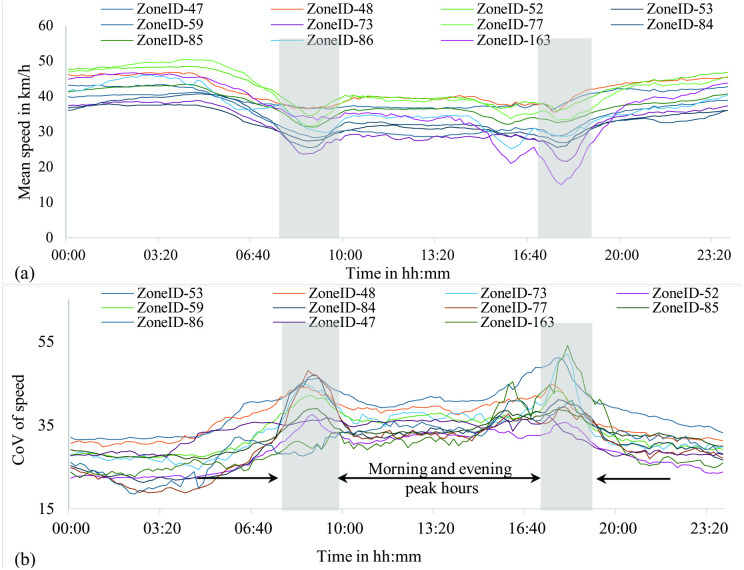
Zonal speed variations across the day and night (a) mean speeds; (b) CoV of speed. This figure compares average traffic speeds and their variability (CoV) across zones throughout 24-hour, illustrating how both central tendency and dispersion differ between day and night.

**Fig 4:**
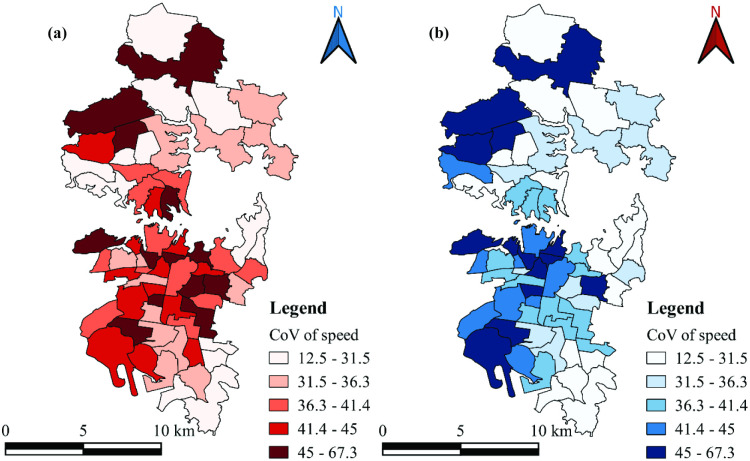
Zonal CoV of speed (a) Morning peak hour; (b) Evening peak hour.

Fig 3 shows the mean speed and CoV of speed for a sample of zones in the study region. It is evident from the figures that speed variation in most zones is more pronounced during specific periods of the day, particularly during morning and evening peak hours. Fig 4 illustrates the spatial distribution of CoV of speed across zones in the study region during both morning and evening peak hours. This figure highlights variations in CoV of speed across zones, with some exhibiting higher speed variations while others demonstrate lower variations. Notably, the speed variations across zones differ between morning and evening peak hours. These differences in CoV across zones during peak hours may be attributed to various factors, including road network connectivity, socioeconomic and travel characteristics, land use patterns, and bus transport connectivity, and this was analyzed through statistical modeling of CS data. Table 3 presents the descriptive statistics corresponding to CS data, road network, public transport, land use, socioeconomic, and travel characteristics. In further sections, a GWR model is developed to identify the factors influencing the speed variability (CoV). Here, the use of CoV allows for a normalized measure of speed variability that is independent of the absolute magnitude of speed [31]. This is particularly useful when comparing across zones with varying mean speed levels. In urban areas like the CBD of Sydney, speed levels can vary significantly between zones due to differences in socio-economic characteristics, landuse patters, local traffic management, travel behavior, etc. In such conditions, CoV enables a fair assessment of variability across such heterogeneous conditions, where the use of mean and standard deviation might underrepresent variability in lower-speed zones and overrepresent it in higher-speed zones.

**Table 3:** Descriptive statistics of CS data and independent variables.

Zonal attributes	µ	σ	Min.	Max.
CS data:				
CoV for morning peak hour	38.28	9.88	12.49	67.28
CoV for evening peak hour	37.08	8.78	12.60	53.44
Road network and public transit attributes:				
Node density (nodes/km^2^)	84.66	44.46	11.56	215.57
Meshedness coefficient	0.36	0.10	0.15	0.67
Completeness	0.00182	0.00073	0.00092	0.00485
Bus stop Density (stops/km^2^)	10.43	7.96	0	29.95
Proportion of Cul-de-sacs	0.14	0.06	0	0.34
Proportion of nodes with 4 or more links	0.14	0.06	0.04	0.31
Road entropy	0.52	0.13	0.19	0.77
Land use attributes:				
Proportion of commercial land	0.084	0.123	0.00	0.543
Proportion of education land	0.035	0.061	0.00	0.438
Proportion of parkland	0.181	0.174	0.000	0.891
Proportion of residential land	0.598	0.232	0.00	0.990
Land use entropy	0.38	0.14	0.03	0.69
Socio-economic characteristics:				
Average monthly household income ($)	2781.10	671.46	0.00	3279.60
Average vehicle ownership per dwelling	1.20	0.43	0.00	2.09
Average age (years)	38.40	6.99	0.00	64.50
Male to Female ratio	0.99	0.22	0.00	2.17
Population density (people/km^2^)	5730.62	4224.34	0.00	18437.51
Vehicle density (vehicles/km^2^)	2440.45	1353.32	0.00	6494.02
Average no. of persons in a household	2.217	0.552	0.00	3.151
Travel characteristics:				
Proportion of public transportation users	0.146	0.082	0.00	0.395
Proportion of private transportation users	0.630	0.194	0.00	1.000
Proportion of other transportation users	0.209	0.121	0.00	0.571

## Modeling the data

In this investigation, GWR models were constructed to ascertain the factors influencing speed variability (CoV). Separate models were developed for morning and evening peak hours. The CoV of CS data corresponding to primary roadways during peak hours on weekdays served as the dependent variable. Independent variables encompassed road network characteristics, socioeconomic attributes, public transit network features, and land use characteristics at a zonal level. The dataset comprised twenty-two independent variables (as shown in Table 3), each representing a distinct dimension. A Principal Component Analysis (PCA) was performed using SPSS to mitigate the dimensionality and multicollinearity (In this study, the Variable Influence Factor was observed to be > 5 for the majority of the variables) and reduce the number of independent variables.

### Principal component analysis (PCA)

PCA is a dimensionality reduction technique commonly employed to reduce the dimensions of independent variables while preserving the maximum variability within the dataset and addressing multicollinearity issues [8,43]. PCA helps identify the underlying structure in the data and summarizes it into orthogonal components. These components are not interpreted in isolation but collectively represent patterns of variation across zones. Therefore, the inclusion of broader variables does not necessarily dilute the interpretability of the results; rather, it enhances the model’s ability to capture multi-dimensional influences in complex urban environments. In this investigation, PCA was conducted with varimax rotation. Prior to PCA, the adequacy of the data and multicollinearity among independent variables were assessed using the Kaiser-Meyer-Olkin (KMO) and Bartlett’s tests. The KMO test statistic for the independent variables yielded a value of 0.640, indicating the dataset’s suitability for PCA [65]. Bartlett’s test revealed the presence of multicollinearity among the independent variables at a significance level of 5%. Then, factor analysis was performed, and the factor loadings were extracted using correlation matrix and varimax rotation. In the analysis, factor loadings with eigenvalues greater than one were retained for further analysis. Subsequently, six-factor loadings were extracted, collectively explaining 81.227% of the total variance in the dataset.

Table 4 presents the rotated factor loadings associated with variables in the dataset. To enhance understanding of the correlation between factor loadings and independent variables, only loadings with an absolute value exceeding 0.4 are included in the table. The choice of factor loading threshold can vary depending on the context and sample size. While a cutoff of 0.6 is commonly recommended for more stringent variable selection, especially in large datasets, several studies have justified the use of lower thresholds (e.g., 0.4 or 0.5) in exploratory factor analysis, particularly when the goal is to retain theoretically or empirically relevant variables that contribute to the overall factor structure [66–68]. Thus, a threshold of 0.4 was employed to strike a balance between statistical robustness and interpretability, ensuring that variables of contextual importance are retained in the analysis. In the factor loadings, higher absolute values signify a stronger correlation with the respective factor, while the sign of the factor loading indicates the direction of correlation. Among the six-factor loadings, factor-1 encompasses seven variables and explains 22.535% of the total variance, and this factor is labelled as “Density”. Factor 2, labelled “Private vehicle dependency,” accounts for 18.864% of the total variance and has five variables. Factor 3, labelled as “Commercial activity,” includes three variables, explaining 12.379% of the total variance. Factor 4, labelled as “Land use diversity and income,” includes three variables and accounts for 10.804% of the total variance. Factor 5, labelled as “Network connectivity,” is loaded with three variables and explains 10.597% of the total variance. Factor 6, labelled “School Zone,” contains only one variable that explains 6.046% of the total variance. Thus, the original variables in the dataset are condensed into six factors, which are subsequently utilized as variables in further regression modelling. The factor scores were computed for each zone using the Anderson-Rubin method, which ensures orthogonality, uncorrelated, and standardized scores [69].

**Table 4:** Rotated factor loadings of independent variables.

Variables	Factor components					
	1	2	3	4	5	6
Vehicle density	0.898					
Node density	0.865					
Population density	0.832					
Bus stop density	0.732					
Proportion of parkland area	−0.629		−0.456			
Proportion of residential area	0.725	0.444				
Proportion of public transport users	0.648					
Proportion of private transport users		0.847				
Average vehicle ownership		0.793				
Average age		0.759				
Average number of persons in household		0.741		0.502		
Road Entropy		−0.503	0.424		0.427	
Male to Female Ratio			0.825			
Proportion of users by other travel modes	0.453	−0.456	0.695			
Proportion of commercial area			0.655			
Completeness index				−0.798		
Average monthly household income	0.436	0.437		0.659		
Land use Entropy				0.655		0.402
Proportion of nodes connected to 4 or more links					0.820	
Meshedness Coefficient		−0.448			0.690	
Proportion of dead ends/ cul-de-sacs		0.472			−0.620	
Proportion of educational area						0.925

## Regression model results

**Fig 5:**
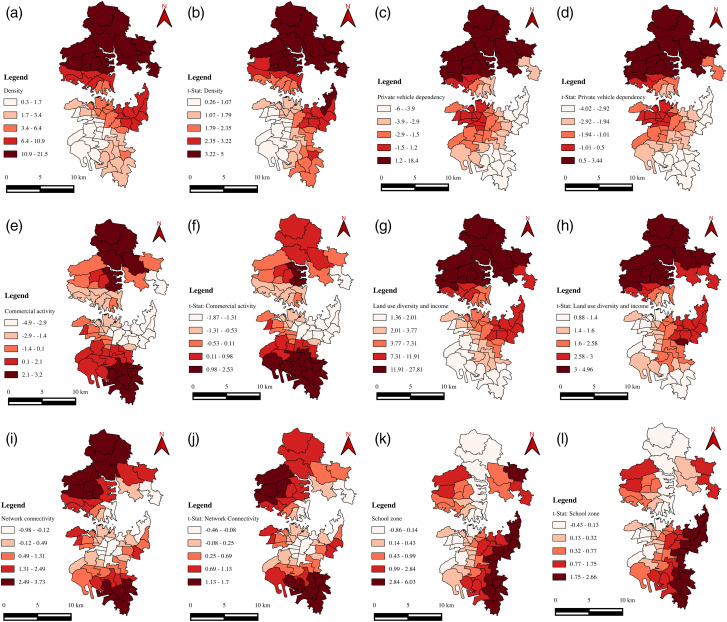
Spatial distributions of local coefficient estimates and corresponding t-statistics for GWR modeling during the morning peak hour. Panels illustrate: (a, b) Density factor; (c, d) Private vehicle dependency; (e, f) Commercial activity; (g, h) Land-use diversity and income; (i, j) Network connectivity; (k, l) School zone variables. This composite figure visualizes both magnitude (coefficient) and statistical significance (t-statistic) of each explanatory variable across spatial zones, highlighting zones where relationships are strong or weak. It underscores the spatial heterogeneity in how these factors influence speed variability.

**Fig 6:**
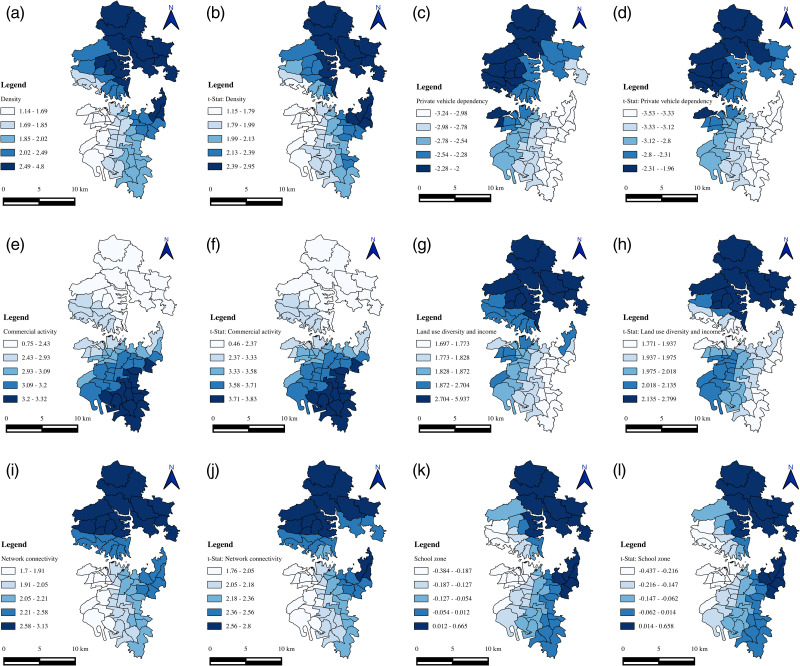
Spatial distributions of local coefficient estimates and corresponding t-statistics for GWR modeling during the evening peak hour. Panels illustrate: (a, b) Density factor; (c, d) Private vehicle dependency; (e, f) Commercial activity; (g, h) Land-use diversity and income; (i, j) Network connectivity; (k, l) School zone variables. This composite figure visualizes both magnitude (coefficient) and statistical significance (t-statistic) of each explanatory variable across spatial zones, highlighting zones where relationships are strong or weak. It underscores the spatial heterogeneity in how these factors influence speed variability.

**Fig 7:**
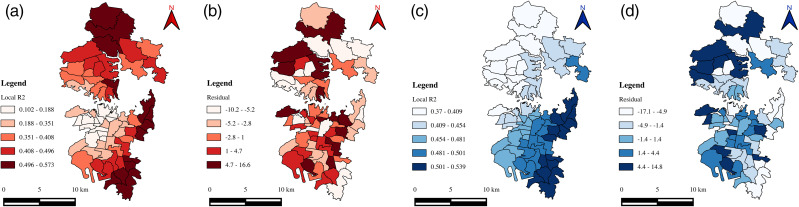
Spatial distribution of local R^2^ values and residuals in Geographically Weighted Regression during peak traffic periods: (a, b) morning peak hour; (c, d) evening peak hour.

Given the study region, it is essential to note that the variables employed are zone-specific and vary across the space (spatial heterogeneity), with each zone possessing its own unique characteristics. Further, the zone-specific characteristics may affect or be affected by neighbouring zones’ characteristics, and this phenomenon is known as spatial autocorrelation. A Moran’s I test was conducted on each independent and dependent variable to detect the presence of spatial autocorrelation. The test revealed significant spatial autocorrelation for most of the variables/factors. Consequently, the global regression models (a single model) based estimates may not be precise for all the zones in the study region [3]. This indicates the necessity of estimating zonal-specific coefficients rather than a single coefficient for the entire study region. Hence, GWR models that account for the spatial autocorrelation were developed using ArcGIS software. These models allow some or all parameters to vary across zones and comprehend the factors/variables influencing speed variability. GWR models were developed to explore the impact of the road network, public transit, land use, and socioeconomic and travel characteristics on speed variability (CoV). Two different models were developed for morning and evening peak hours, respectively. The summary statistics of the two models (local regression) are presented in Tables 5 and 6. Figs 5–7 depict the spatial distribution of variable coefficients and their t-statistics, model residuals, and local *R*^*2*^ for morning and evening peak hours.

**Table 5:** Summary of GWR model for morning peak hour.

Variables	Mean	S.D	Min	Max
Intercept	36.63	4.17	25.92	40.795
Density	6.28	5.493	0.33	21.47
Private vehicle dependency	−0.143	6.1	−5.95	18.44
Commercial activity	−0.56	3.28	−4.93	3.19
Land use diversity and income	7.97	7.135	1.36	27.81
Network Connectivity	1.07	1.27	−0.98	3.73
School Zone	1.39	1.66	−0.86	6.03
*R* ^*2*^	0.64			
*Adj. R* ^*2*^	0.43			
AICc	468.63			
Mean square error (MSE)	38.998			
Standard error	6.245			

*S.D = Standard Deviation.*

**Table 6:** Summary of GWR model for evening peak hour.

Variables	Mean	S.D	Min	Max
Intercept	37.07	0.62	34.86	38.28
Density	2.17	0.74	1.14	4.80
Private vehicle dependency	−2.64	0.34	−3.24	−2.00
Commercial activity	2.77	0.61	0.75	3.32
Land use diversity and income	2.35	1.04	1.70	5.94
Network Connectivity	2.23	0.37	1.70	3.13
School Zone	−0.05	0.19	−0.38	0.66
*R* ^*2*^	0.50			
*Adj. R* ^*2*^	0.40			
AICc	439.79			
Mean square error (MSE)	42.875			
Standard error	6.548			

### Morning peak hour model

In the morning peak hour, the spatial distribution of the coefficients exhibits considerable variation across the study region, indicating spatial heterogeneity in the influence of explanatory variables (Table 5). However, the statistical significance of these factors is generally weak across the region. The *Adj. R²* value for this model is 0.43, suggesting that approximately 43% of the variability in speed can be explained by the selected factors. The density factor presents predominantly positive coefficients (Fig 5a), implying that speed variability increases with higher density levels. Nevertheless, the *t-*statistics (Fig 5b) suggest that these coefficients lack statistical significance at the 0.10 level across many zones. The private vehicle dependency factor (Fig 5c) displays a mixed pattern, with coefficients shifting from negative to positive across the region, and *t-*statistics (Fig 5d) indicating statistical insignificance throughout. Similarly, the commercial activity factor (Fig 5e) demonstrates a transition in sign from negative to positive, reflecting inconsistent influence on speed variability, with only 30–40% of the zones showing statistically significant coefficients at the 0.10 level (Fig 5f). The land use diversity and income factor (Fig 5g) shows positive coefficients across the study region, ranging from 1.36 to 27.81, suggesting increased variability with greater land use diversity and income. However, statistical significance is observed in only about 60% of the zones (Fig 5h). The network connectivity factor (Fig 5i) ranges from −0.98 to 3.73, with both negative and positive values, but none of the coefficients are statistically significant (Fig 5j). Lastly, the school zone effect is found to be statistically insignificant across all zones, with coefficients ranging from −0.86 to 6.03. The spatial distribution of local *R²* values (Fig 7a) ranges from 0.102 to 0.57, with stronger model fit observed in the northern and eastern parts of the study area and weaker performance near the Central Business District (CBD).

### Evening peak hour model

In contrast to the morning peak hour, the evening peak hour model presents a more spatially consistent and statistically robust explanation of speed variability (Table 6). Although the *Adj. R²* is marginally lower than the morning peak model; the explanatory power of the modeled factors is more coherent across the region. The density factor (Fig 6a) yields positive coefficients, with values ranging from 1.14 to 4.80. The corresponding *t-*statistics (Fig 6b) reveal that these coefficients are statistically significant at the 0.10 level across nearly all zones. The private vehicle dependency factor (Fig 6c) consistently exhibits negative coefficients across the region, with significant *t-*statistics (Fig 6d), indicating a meaningful inverse relationship with speed variability. The commercial activity factor (Fig 6e) consistently shows positive coefficients, suggesting increased speed variability with higher levels of commercial activity. The *t-*statistics (Fig 6f) confirm statistical significance for nearly all zones in the region. The land use diversity and income factor (Fig 6g) also displays positive coefficients, ranging from 1.70 to 5.94, with statistical significance observed across the study region (Fig 6h). The network connectivity factor (Fig 6i) consistently yields positive coefficients (1.70 to 3.13), with all zones demonstrating statistical significance as indicated in Fig 6j. Similar to the morning model, the school zone factor remains statistically insignificant, with coefficients ranging from −0.38 to 0.66. Local *R²* values for the evening peak hour model (Fig 7c) range from 0.37 to 0.54. Notably, the model demonstrates a gradual increase in explanatory strength from the northern to the southern zones of the study area, reflecting spatial continuity in model performance.

### Models comparison

Comparing both models reveals several important contrasts. The distribution of coefficients for the variables in the morning peak hour is relatively widely spread across the study region. Further, none of the variables in the morning model consistently exhibit statistical significance across the entire study region, and many factors show mixed or insignificant effects. While the morning peak hour model exhibits slightly higher global *Adj. R²* values, the spatial variability, and inconsistency in coefficient significance limit its interpretability. In contrast, the evening peak hour model demonstrates stronger coherence and statistical reliability. Except for the school zone factor, all other variables are statistically significant across the region, and the direction of the coefficients is consistent with theoretical expectations. Furthermore, the Akaike Information Criterion corrected (AICc) values also indicate superior model performance for the evening peak hour, suggesting its greater explanatory capability in capturing the determinants of speed variability. In summary, while both models offer insights into spatial variability in traffic speed, the evening peak hour model is more robust and reliable in explaining the observed patterns, thereby underscoring its suitability for informing traffic management and policy strategies.

## Discussion

Analysing traffic speed variability is critical for evaluating both operational efficiency and roadway safety. This study examined the impact of zonal-level characteristics corresponding to road network, public transit, land use patterns, socioeconomic, and travel on traffic speed variability during morning and evening peak periods. The findings indicate that the evening peak hour model was more effective and consistent in explaining speed variability, with Local *R*^*2*^ values ranging from 0.37 to 0.54 (Fig 7c). Furthermore, the Local R^2^ values exhibit a gradual increase from the northern to the southern zones of the study area, indicating a clear spatial continuity in model performance. In contrast, the morning peak hour model exhibits Local R^2^ values ranging from 0.102 to 0.57 (Fig 7a), with stronger model fits scattered across different parts of the study area, thereby indicating spatial discontinuities in model performance. The potential reason for morning peak hour model having a better R^2^ as compared to the evening peak hour could be attributed to the differences in spatial patterns of the response variable across the zones. The higher R^2^ but non-significant predictors in the morning peak model suggest that traffic behavior during this period is spatially uniform and influenced by strong directional patterns, leading to a stable yet statistically indistinct relationship among variables. Conversely, the lower R^2^ but significant predictors in the evening model indicate greater spatial heterogeneity and local variation in travel behavior, allowing the predictors to exhibit stronger localized effects even though the overall explanatory power of the model decreases. Supporting this, a study by Wemegah et al., [70] found that traffic during peak and off-peak hours is inconsistent across weekdays. Further, the evening peak hour model was found to be more significant than the morning peak. Similarly, Pan et al., [3] compared the traffic states between morning and evening peak periods on weekdays. In light of higher traffic during the evening peak hour, the authors investigated the effect of the built environment only on the traffic state during the evening peak hour. In the present study, the average speed for the evening peak was comparatively lower than that of the morning peak (Fig 3a). Further, the variations in speed (CoV) across the zones for the evening peak are high compared to the morning peak. Given these observations, the relevance of each factor in the evening peak hour model is discussed.

Spatially, the density factor is positively associated with the dependent variable (Fig 6a), indicating that as density increases, speed variability on primary roads during evening peak hours increases. Density typically reflects the degree of compactness within a given area or volume. Here, the factor represents the concentration of vehicles, characteristics of the road network, population levels, public transport availability, and land-use patterns in each zone. The spatial distribution of *t-*statistics (Fig 6b) confirms the statistical significance of the estimated coefficients. The factor has stronger influence in northern and eastern parts of the study region. The findings of this factor align with many earlier studies [3,24,31,35,36,54,71]. Parthasarathi found that traffic congestion increases with population density [54]. A study by Li et al., found that under dry weather conditions, speed variability increases with vehicle density [31]. Wang et al., and Kumar et al., found that more bus stops are associated with increased speed variation [24,71]. In the case of traffic volume and bus stop density, Pan et al., found a positive relationship with the traffic state index [3]. Another study by Nian et al., found that bus stop density and residential community are some of the key factors affecting roadway speed [35]. Interestingly, Martinelli et al., found a positive relationship between the bus stop density and mean speed, and a negative relationship between bus stop density and standard deviation of speed [36]. Zhong et al., also found that the number of bus stops positively correlates with average speed, possibly due to exclusive bus lanes minimising the impact on actual traffic speed [34].

The coefficient of the private vehicle dependency factor exhibits a consistent negative sign across the study region (Fig 6c). This factor represents the characteristics of trip makers such as average vehicle ownership, age, and number of persons in the household. The t-statistics (Fig 6d) suggest significance in explaining speed variability across the study region. Ideally, one would expect a positive impact on this factor. In spite of the statistical significance of the relation, a further investigation of these variables is necessary to understand more about its effect on traffic speed. The commercial activity factor coefficient consistently shows a positive sign across the study region (Fig 6e). The t-statistics (Fig 6f) show that the estimated coefficients are statistically significant across the study region. This factor encompasses variables related to commercial and business activities. A plausible explanation for the observed relationship is that zones with higher commercial intensity typically experience a greater presence of the male workforce and a wider variety of transport modes used for the movement of goods and services. During evening peak hours, such zones are likely to attract increased trip generation related to work, education, recreation, and shopping. This finding aligns with previous studies. Pan et al., found that public and commercial buildings positively affect traffic states during evening peak hours [3].

The coefficient of land use diversity and income factor is positive (Fig 6g) across the entire study region. This factor represents the land use entropy, average monthly household income, and completeness index variables (Table 4). The positive relationship indicates that zones with higher land-use diversity and income tend to show greater temporal fluctuations in operating speeds. This is primarily because such zones experience complex travel behaviour and higher variability in traffic demand, particularly during evening peak periods. The coefficient for the network connectivity factor is positive and statistically significant (Figs 6g & h) across the entire study region. According to Table 4, this factor is associated with the proportion of nodes connected to more than or equal to 4 links, the meshedness coefficient, and the proportion of cul-de-sac variables. The plausible reason for a positive relationship is that, in the road network, the nodes with more links often handle higher traffic demand, leading to heavy traffic and greater speed variability. In contrast, cul-de-sacs reduce the number of intersections and access points on primary roads, leading to fewer instances of vehicles entering and exiting traffic flow. This might result in smoother and more consistent traffic speeds on primary roads. In this line, the findings align with previous studies on travel time [8]. The coefficients associated with the school zone factor exhibit a consistent negative trend across the entire study region, i.e., as the school zone increases, the speed variability decreases. This factor represents the proportion of educational areas. To ensure the safety of children and students, speed limits are often reduced in educational areas such as schools and colleges located on primary roads. These lower speed limits may lead to more consistent, lower speeds, resulting in decreased speed variability. However, from the *t-*statistics (Fig 6l), the estimated coefficients are insignificant in explaining the dependent variable. Therefore, further investigation is necessary to confirm this relationship.

In summary, the evening peak hour model results are consistent with findings from earlier studies [3,31,35,54,71], while the results are inconsistent from other studies [34,36]. The factors density, private vehicle dependency, commercial activity, land use diversity and income, network connectivity are significant in explaining the variability. Thus, GWR proved effective in capturing local variations in the relationships between urban form, travel behavior, and traffic performance particularly during the evening peak. The model outcomes demonstrate the spatial heterogeneity of underlying factors, reinforcing the advantage of localized regression frameworks for traffic research and planning. However, the model fits cannot be compared with the other studies as it mainly depends on the study scopes [44]. In contrast to understanding the factors influencing speed variability, several studies emphased on investigating the macroscopic factors including traffic parameters at zonal level or city level on the crash frequency [39,40,44]. Phan and Truong investigated the effect of traffic congestion on total crashes, fatal or serious injury crashes and fatal only crashes in both morning and evening peak hours [39]. The study found that traffic congestion tends to increase the total crashes but decreases the fatal crashes in both the peak hours. Another study by Wang et al., and Hern´andez explored the local associations between crash frequency and road network attributes, socio-economic characteristics and land use factors [44,45]. The analysis revealed that road network factors exhibited high importance, while socio-economic variables exhibited moderate effects and the land use variables showed lower effects. Xiao et al., highlighted the significance of incorporating the traffic varibles in understanding the variations in crash frequency [40]. Together, these findings reinforce the importance of applying localized spatial modeling techniques in transportation research whether for speed variability or crash frequency to uncover nuanced patterns that global models may overlook.

## Conclusion

Speed is an important metric to assess road network performance and safety. Numerous studies have been conducted to understand the roadside and on-road features influencing traffic speed. Several studies have also tried to assess the effect of road network characteristics on speed variations. However, there has been limited research on the spatial variations of speed at the zonal level. Understanding these spatial variations can significantly affect land use and city planning. Additionally, analysing speed performance and reliability at the zonal level can impact the real estate market, as residents will likely prefer zones with better traffic reliability. In this study, CS data, collected every 10 minutes over a month in 2018, was analysed to understand speed variability. Given the data availability and the significance of road hierarchy (as shown in Fig 2), the analysis was confined to primary roads and weekdays only. Preliminary analysis revealed that the mean speed and speed variation (CoV) in the majority of the zones is more significant during specific periods of the day, i.e., during morning and evening peak hours. Variations in CoV across zones during peak hours may be influenced by factors such as road network connectivity, socio-economic characteristics, land use patterns, and bus transport connectivity. These factors were analysed using statistical modelling of the CS data.

Two different models were developed, i.e., one for the morning peak and another for the evening peak hour. The original dataset contained 22 independent variables corresponding to road network connectivity, socio-economic and travel characteristics, land use patterns, travel characteristics, and public transport details. Many of these variables had a Variance Inflation Factor (VIF) greater than 5, indicating multicollinearity. Therefore, PCA was performed to address the issues of a large number of independent variables, multicollinearity, and dimensionality. Six-factor loadings were extracted from the PCA, explaining 81.227% of the total variance in the original dataset. Further, Moran’s I test confirmed the presence of spatial autocorrelation. Considering these aspects, GWR models were developed to understand the factors influencing zonal speed variability. The CoV of speed was modelled as a function of six factors. The results showed that, for the morning peak hour, none of the factors is consistent in logically explaining the variability of the dependent variable across the study region. Meanwhile, in the evening peak hour, density, private vehicle dependency, commercial activity, land use diversity and income, and network connectivity factors logically explain the variability across the study region. Among these, four factors have shown a positive impact, while one has shown a negative impact on the dependent variable. On the practical implementation point of view, it is suggested that urban planners should prioritize land use strategies that balance residential, commercial, and institutional zones. This specifically refer to planning approaches that reduce extreme clustering of single land use types and instead promote mixed-use development. For example, integrating small-scale commercial or institutional facilities within predominantly residential areas can reduce long-distance commuting, thereby mitigating peak-hour traffic concentrations and speed variability. With regard to the recommendation of zone-specific interventions, our analysis indicates that the determinants of speed variability differ across zones, as shown in the geographically weighted regression results. This spatial heterogeneity implies that a uniform city-wide strategy may overlook localized factors that strongly influence speed patterns (e.g., high commercial intensity in one zone versus predominantly residential character in another). Therefore, interventions tailored to the land use and traffic characteristics of individual zones would be more effective than a one-size-fits-all approach. However, further investigation is needed to confirm the relationships of some variables.

This is one of the very few studies that have explored the zonal variables and found that they significantly impact speed variability. Therefore, along with the microscopic characteristics, roadside and on-road features, macro/mesoscopic characteristics corresponding to the road network, land use, socioeconomic, and travel at the zonal level also play a significant role in speed variability. However, this study has some limitations. Considering the data availability, the analysis was only confined to primary roads and only for a small portion of the city. In the future, CS data may be collected for a complete city on all the important road hierarchies to develop and implement strategies to improve road network performance and safety. Another notable limitation of this study is that the analysis is based on traffic and associated zonal-level data collected in 2018. Given the pace of urban development, technological advancements in transport systems, and evolving travel behavior over recent years, the findings may not fully capture current conditions. Future studies should aim to incorporate correction factors or develop macro models that adjust for temporal changes. Further, similar studies must be performed on other cities located across the world to understand the inherent functionalities of different urban forms on road network performance and safety.

## Supporting information

S1 File Aggregated speed data supplimentary material. (XLSX)

S2 File Data for GWR. (XLSX)

S3 File EPH_GWR_Output. (XLSX)

S4 File MPH_GWR_Output. (XLSX)
